# Ventilation

**Published:** 1880-03

**Authors:** 


### VENTILATION.

"We all seek health and comfort and yet are neglectful of the means to obtain them, and are willing oftentimes to substitute what is very imperfectly adapted to the purpose and continue its use from habit, rather than avail ourselves of a better way; there is no one thing in the routine of daily life which so much contributes to both health and comfort, as a thoroughly ventilated dwelling; it keeps the air of home fresh, gives a genial tone to the spirits, keeps us wide awake, the muscular system in a healthy state of tension, imparts zest to an appetite, making the preparations of the table more palatable, protects the nervous system from all unpleasant and uncomfortable draughts of air and prevents taking cold, maintains a uniform circulation of the blood, does not overtax the lungs, promotes ready and regular digestion, suggests a pleasant word instead of a fretful one, makes sleep sweet and refreshing, and even infuses into dreams an halo of peace, allows greater scope and activity to the imagination, a clearer action to our ideas, assists the judgment in matters pertaining to its exercise, in fact affects the well-being of body and mind in all their functions; all this will be found true upon careful thought on this matter, pure air is the very life of all things. The manner and ways by which we are affected by thorough ventilation, or no ventilation at all, are numberless; the latter, every year, sends more to the grave than the victims or their friends are aware of; disease preys more actively on the constitution under this condition of bad air and may be easily communicated, whereas under good ventilation they would be checked; more colds are brought on by bad air indoors, than are taken out-doors; a majority of the colds under which people suffer in winter can be traced directly to the condition of the air in-doors, and rarely can one be attributed to the state of the air out-doors, whether it be rain or shine, hot or cold; not that colds always originate by breathing impure or bad air, but such air frequently, yes always aggravates a cold, and often establishes a slight cold upon the system, which otherwise would not have become fixed, stubborn or fatal. With all the advantages on the side of thorough ventilation and all the dangers and discomforts on the other, of non-ventilation, who would not choose that the former condition should be that of the house where he resides, instead of the latter. Many times there is a feeling of lassitude, sleepiness, dullness or stupidity, which is attributed to the state of health, when it is really the air we are breathing which lacks vitality; living in a house illy ventilated, always containing more or less of vitiated air, will more or less, sooner or later, affect the state of health, a too hearty dinner is made much more injurious by remaining in-doors where the air is not pure, than by going out of doors where the air is pure, although immediate exercise after dinner should be avoided. In the summer there is no difficulty in getting the best of ventilation, but in cold weather when fires are in request, then some system of ventilation becomes imperatively necessary, if our health and comfort would be properly protected and cared for. In this climate many attempts have been made, and large sums expended, to attain to a thorough and perfect ventilation, but they have heretofore been only partial and very imperfect, and whenever ventilation has been accomplished it has been done in an imperfect manner at best, and at a *heavy* additional cost for fuel, for the reason that *the demands of a perfect and thorough ventilation have never been complied with.* What is required for the complete and thorough ventilation of a dwelling house? We must go to nature herself, and enquire, and she will answer, three things are requisite; the same which we find existing out of doors. In the open air there we find the air to be constantly in motion, we also find it to be constantly changing, we also find it to be uniform in temperature whether it be warm or cold; make these the conditions of the air indoors and there must be perfect and thorough ventilation, no matter what may be the means by which these conditions are effected or produced. In order then to have perfect and thorough ventilation indoors we must, 1st, keep the air in constant motion, 2d, make the air to be constantly changing, 3d, have the air equalized and made uniform in temperature. When these requirements are complied with then we shall have our dwellings ventilated in the best and most perfect manner."

